# Modulation of extracellular matrix in cancer is associated with enhanced tumor cell targeting by bacteriophage vectors

**DOI:** 10.1186/s12943-015-0383-4

**Published:** 2015-06-03

**Authors:** Teerapong Yata, Eugene L. Q. Lee, Keittisak Suwan, Nelofer Syed, Paladd Asavarut, Amin Hajitou

**Affiliations:** Phage Therapy Group, Division of Brain Sciences, Department of Medicine, Imperial College London, Hammersmith Hospital Campus, Burlington Danes Building, London, W12 0NN UK; National Nanotechnology Center, National Science and Technology Development Agency, 111 Thailand Science Park, Pathumthani, 12120 Thailand; The John Fulcher Molecular Neuro-Oncology Laboratory, Division of Brain sciences, Imperial College London, Hammersmith Hospital Campus, Burlington Danes Building, London, W12 0NN UK; Amin Hajitou, Burlington Danes Building, Hammersmith Hospital Campus, 160 Du Cane Road, London, W12 0NN UK

**Keywords:** Extracellular matrix, Cancer gene therapy, Multicellular tumor spheroids, Bacteriophage

## Abstract

**Background:**

Gene therapy has been an attractive paradigm for cancer treatment. However, cancer gene therapy has been challenged by the inherent limitation of vectors that are able to deliver therapeutic genes to tumors specifically and efficiently following systemic administration. Bacteriophage (phage) are viruses that have shown promise for targeted systemic gene delivery. Yet, they are considered poor vectors for gene transfer. Recently, we generated a tumor-targeted phage named adeno-associated virus/phage (AAVP), which is a filamentous phage particle whose genome contains the adeno-associated virus genome. Its effectiveness in delivering therapeutic genes to tumors specifically both *in vitro* and *in vivo* has been shown in numerous studies. Despite being a clinically useful vector, a multitude of barriers impede gene transduction to tumor cells. We hypothesized that one such factor is the tumor extracellular matrix (ECM).

**Methods:**

We used a number of tumor cell lines from different species and histological types in 2D monolayers or 3D multicellular tumor spheroid (MCTS) models. To assess whether the ECM is a barrier to tumor cell targeting by AAVP, we depleted the ECM using collagenase, hyaluronidase, or combination of both. We employed multiple techniques to investigate and quantify the effect of ECM depletion on ECM composition (including collagen type I, hyaluronic acid, fibronectin and laminin), and how AAVP adsorption, internalisation, gene expression and therapeutic efficacy are subsequently affected. Data were analyzed using a student’s *t* test when comparing two groups or one-way ANOVA and *post hoc* Tukey tests when using more than two groups.

**Results:**

We demonstrate that collagenase and hyaluronidase-mediated degradation of tumor ECM affects the composition of collagen, hyaluronic acid and fibronectin. Consequently, AAVP diffusion, internalisation, gene expression and tumor cell killing were enhanced after enzymatic treatment. Our data suggest that enhancement of gene transfer by the AAVP is solely attributed to ECM depletion. We provide substantial evidence that ECM modulation is relevant in clinically applicable settings by using 3D MCTS, which simulates *in vivo* environments more accurately.

**Conclusion:**

Our findings suggest that ECM depletion is an effective strategy to enhance the efficiency of viral vector-guided gene therapy.

## Background

Cancer is a complex genetic disease that results in malignancy of native tissue [1]. Gene therapy was initially conceived as an approach for treating genetic diseases; however, its scope has expanded to include treatments for cancer [2]. Currently, over 60 % of ongoing clinical gene therapy trials are designed to treat cancer [3]. Most cancer gene therapy vectors are invasively administered via intratumoral injections, and the development of non-invasive systemic vectors are greatly warranted. In addition to potentially causing less harm to the patient, it may target both localized and metastatic tumors. Successful cancer gene therapy depends on the development of vectors able to deliver therapeutic genes to tumors specifically and efficiently, while sparing healthy tissues. Animal viruses have the potential to be developed for targeted gene transfer, but require elimination of their native tropism for mammalian cells [4]. This approach however, is challenging because engineering viruses to target non-natural receptors greatly reduce their efficiency [5].

We have previously reported the generation of a hybrid bacteriophage vector for targeted systemic cancer gene therapy and molecular imaging [6]. This construct, termed (adeno-associated virus/phage) AAVP, is a combination of a mammalian transgene cassette flanked by inverted terminal repeats (ITRs) from adeno-associated virus 2 (AAV2) and a fUSE-5 (peptide display) vector derived from the fd bacteriophage genome. Phages have evolved to infect bacteria only and thus, unlike eukaryotic viruses, have no strategies to deliver genes to mammalian cells. The AAVP displays the cyclic RGD4C (CDCRGDCFC) fusion peptide on its pIII capsid protein, allowing homing to and entry via αv integrin receptors such as α_v_β_3_ and α_v_β_5_, which are expressed by cancer cells or cancer-associated endothelial cells, but not on normal tissues or vasculature [7, 8]. Moreover, the RGD4C.AAVP construct has allowed for drastic improvements in gene delivery rates to cancer cells over conventional bacteriophage vectors, as shown in numerous *in vitro* and *in vivo* studies, including a large-scale cancer trial involving pet dogs with natural cancers [9]. Even though the targeting and efficiency of the RGD4C.AAVP has improved with the modifications applied thus far, there still exists a large room for improvement.

An important consideration is not all limitations are attributable to the vector. Cancer cells in particular, possess macro- and microanatomical barriers that impede gene delivery. Specifically, desmoplastic reactions result in substantial extracellular matrix (ECM) formation around tumors, cancer-associated fibroblasts and infiltrating immune cells [10]. The resultant high interstitial fluid pressure (IFP), spatial hindrance and inhibition of cell-surface receptors decrease uptake of therapeutics [11]. As such, depletion of the ECM before administration of therapeutics constitutes a mechanism for tumor priming [12]. ECM clearance should allow increased transport and binding of RGD4C.AAVP to αv integrin receptors on the tumor cell surface. This principle of transduction has already been demonstrated in multiple studies through the use of ECM-depleting enzymes [13–15].

We sought to test the hypothesis that ECM depletion can increase the tumor transduction efficacy of RGD4C.AAVP vectors by evaluating the effects of co-administering AAVPs after treatment of cancer cells with collagenase, hyaluronidase or a combination of both. Our results show that ECM degradation is a powerful adjuvant in raising transduction rates for phage-guided cancer therapy. These findings were further verified through RGD4C.AAVP-mediated cancer killing by delivering the conditionally toxic Herpes simplex virus-thymine kinase (*HSVtk*) suicide gene in conjunction with ganciclovir treatment. We have also validated this strategy through a multicellular tumor spheroid model (MCTS). Our results demonstrate how modulating the tumor microenvironment may enhance the efficacy of RGD4C.AAVP and other gene delivery vectors as powerful implements against cancer.

## Results

### Treatment of tumor cells by collagenase and hyaluronidase affects tumor ECM composition

Firstly, it is important to determine the composition of ECM constituents after treatment of tumor cells by collagenase, hyaluronidase, or a combination of both. We selected three candidate tumor cell lines − 9L rat gliosarcoma, as well as human MCF-7 breast cancer and human LNCaP prostate cancer for evaluation.

**Fig. 1:**
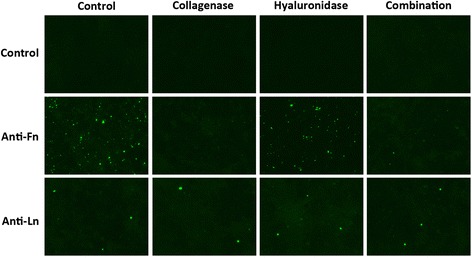
Immunostaining of fibronectin and laminin in LNCaP cells after ECM depletion. LNCaP cells were treated with collagenase, hyaluronidase or a combination of both enzymes before incubation with mouse anti-fibronectin (Anti-Fn) or mouse anti-laminin (Anti-Ln) antibodies, followed by goat anti-mouse AlexaFluor 488-conjugated secondary antibody. Depletion of fibronectin after treatment with collagenase or a combination of both enzymes, but not with hyaluronidase alone was clearly observed. No observable differences could be seen for laminin. Experiments including secondary antibody alone were used as negative controls. Data shown are representative of all three cell lines tested (LNCaP, 9L and MCF-7). Experiments were repeated twice and individual conditions were repeated in triplicates

Qualitative analysis of changes in fibronectin and laminin post-enzymatic treatment was performed by immunostaining using anti-fibronectin or anti-laminin antibodies. We consistently observed a significant decrease in fibronectin expression, but not laminin, post-collagenase or collagenase plus hyaluronidase treatments of LNCaP cells (Fig. 1), as well as 9L and MCF-7 cells (data not shown). Conversely, we observed no differences in fluorescence of cells after treatment with hyaluronidase alone, indicating that the decrease in fibronectin, as indicated by fluorescence, is attributable to collagenase treatment (Fig. 1).

**Fig. 2:**
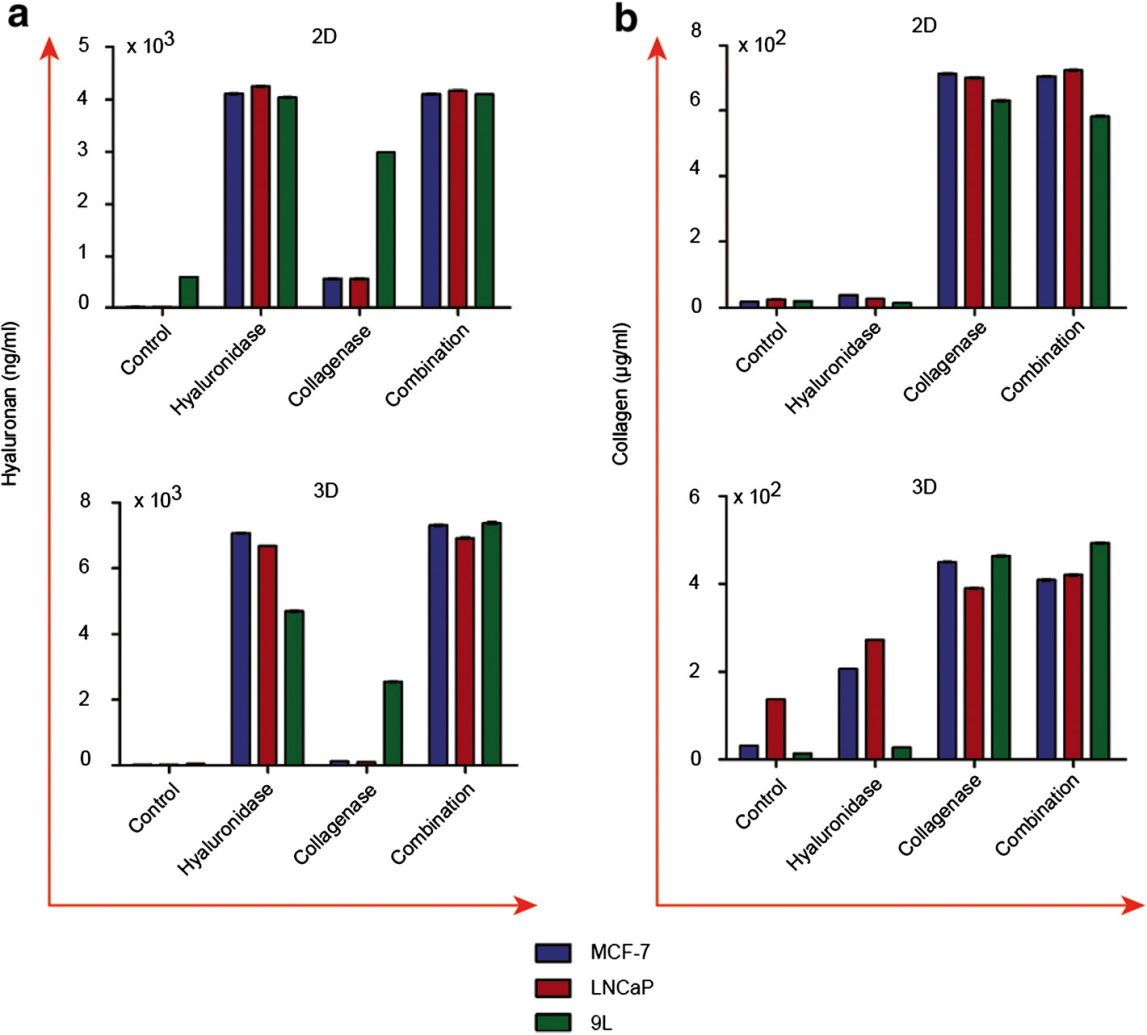
Hyaluronic acid ELISA and Collagen Sirius red assay of cell culture media after collagenase and hyaluronidase treatments of 2D cell monolayers and MCTS of MCF-7, LNCaP and 9L. Cell monolayers or MCTS were treated with hyaluronidase or collagenase, or combination of both enzymes before the culture supernatants were analysed for hyaluronic acid (hyaluronan, HA) or collagen (type I). **a** Hyaluronic acid ELISA was based on adding biotinylated HA binding proteins to a plate coated with umbilical cord HA and subsequent treatment with peroxidase-conjugated anti-biotin antibodies. The results, show release of HA in to the culture medium primarily after treatment with hyaluronidase or a combination of both enzymes. **b** Sirius Red assay (chondrex) results showing release of collagen in to the culture medium primarily after treatment with collagenase or a combination of both enzymes. Experiments were repeated twice and individual conditions were repeated in triplicates. Data are presented as mean ± s.e.m

Moreover, we quantified the presence of type I collagen and hyaluronic acid (hyaluronan, HA) in the supernatants of cells after treatment with collagenase, hyaluronidase or both using colorimetric assays in 2D cell monolayers and 3D MCTS. HA levels were quantified by an optimized ELISA protocol for HA detection using biotinylated HA binding proteins (Fig. 2a). Collagen was quantified using a Sirius Red assay before and after enzymatic treatment (Fig. 2b).

After treatment with hyaluronidase or combination of hyaluronidase and collagenase, the concentration of HA in the supernatant was 4000-4025 ng/ml for 2D monolayer and 6673-7378 ng/ml for 3D MCTS, excluding 9L MCTS, which released only 64 % of HA relative to combined treatment (Fig. 2A). Interestingly, only 9L cells released relatively higher levels of HA after treatment with collagenase alone in both 2D and 3D cultures (2987 ng/ml and 2551 ng/ml, respectively). In 2D cultures however, MCF-7 and LNCaP cells both released 552 ng/ml HA after treatment with collagenase, but not in 3D cultures.

The reported concentration of collagen in the supernatant of MCF-7, LNCaP and 9L cells ranged between 580-724 μg/ml for 2D monolayer cultures and 390-493 μg/ml for the 3D MCTS following treatments with collagenase or combination of collagenase plus hyaluronidase, as compared to baseline measurements (Fig. 2B). Baseline collagen concentrations after treatment with hyaluronidase were negligible for 2D monolayers; however, in 3D MCTS, MCF-7 and LNCaP cell lines increased collagen release in to the supernatant, 207 and 272 μg/ml, respectively, upon hyaluronidase treatment alone.

### Removal of ECM proteins enhances diffusion and internalization of RGD4C.AAVP

After establishing that enzymatic treatment depletes specific ECM constituents, we aimed to investigate how it may affect diffusion and cell internalization of RGD4C.AAVP. We performed a RGD4C.AAVP diffusion assay and assessed the effect of ECM depletion on RGD4C.AAVP internalization in 9L rat gliosarcoma cells.

**Fig. 3:**
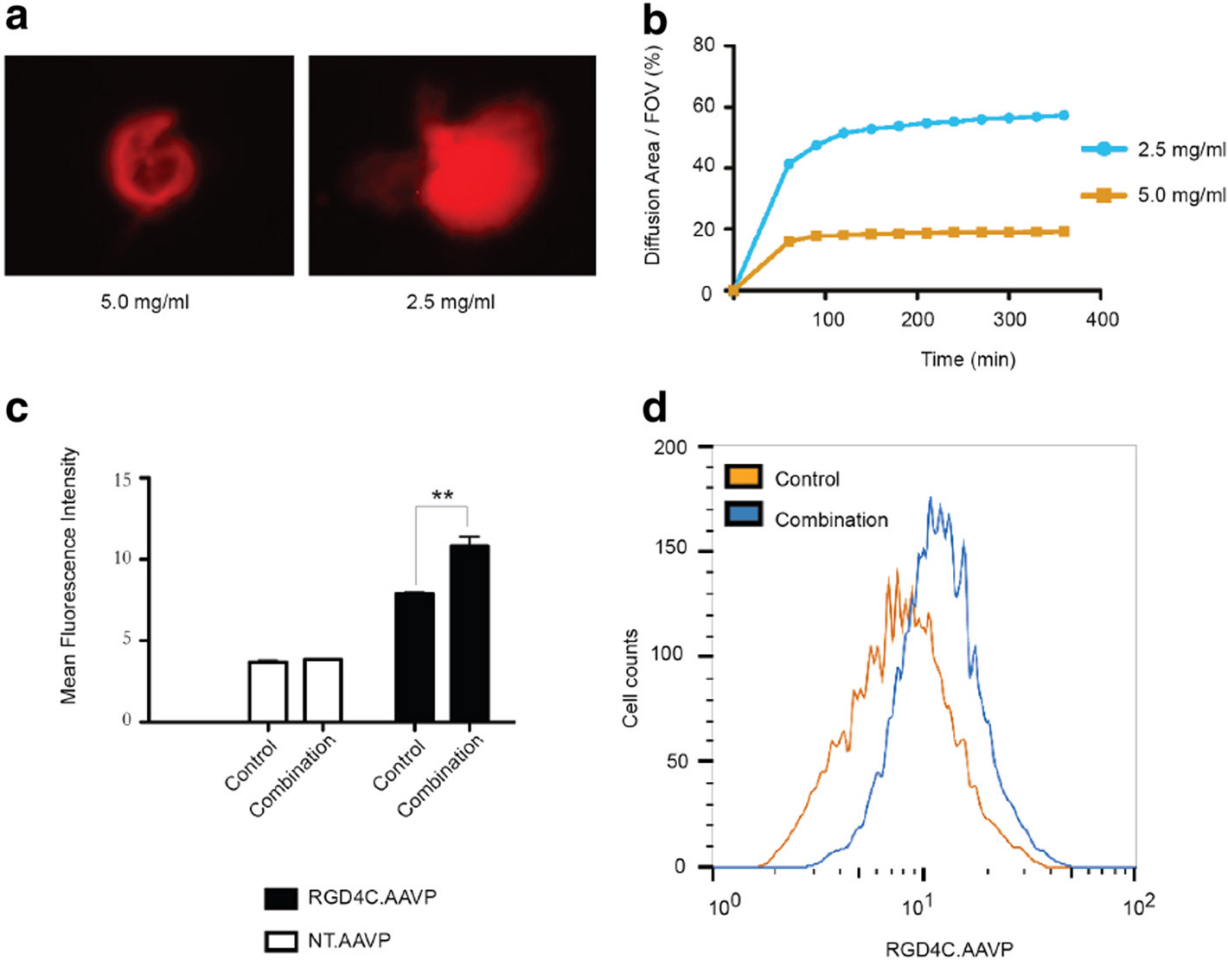
Cellular diffusion and internalization of RGD4C.AAVP is boosted by ECM clearance. **a** RGD4C.AAVP diffusion assay was carried out using fluorescently-labelled RGD4C.AAVP and followed through an ECM gel matrix. Two ECM concentrations were tested (2.5 and 5.0 mg/ml) and RGD4C.AAVP was tracked using fluorescent microscopy. **b** Measurement of RGD4C.AAVP diffusion, per fields of view (FOV), at various time points for a total time of 6 h. **c** Flow cytometric analysis of AAVP uptake was performed in 9L cells after ECM depletion. Cells were treated either with a combination mix of collagenase (0.2 mg/ml) and hyaluronidase (0.4 mg/ml) or without any enzyme (control) for 1 h before incubation with either non-targeted NT.AAVP (white bars) or RGD4C.AAVP (black bars). Fixation was conducted 4 h post-transduction and immunofluorescence was performed using rabbit anti-phage primary and goat anti-rabbit AlexaFluor-647 conjugated antibodies. Gating threshold was set at 10,000 events of total cell population. Each condition was measured in triplicate and statistics were obtained by unpaired student *t*-test (** = *p* < 0.01). Data are presented as mean ± s.e.m. **d** The graph shows shifts in mean fluorescence intensity and RGD4C.AAVP positive cell counts between no-enzyme control (yellow line) and enzyme combination treatment (blue line). Experiments were repeated twice and individual conditions were repeated in triplicates

We investigated the movement of RGD4C.AAVP through ECM matrices at different concentrations by using confocal microscopy, and observed that fluorescently-labeled RGD4C.AAVP particles had greater diffusion when introduced into lower concentrations of ECM-gel matrix compared to higher concentrations (Fig. 3a). Fluorescently-tagged RGD4C.AAVP moved through the 2.5 mg/ml ECM 2.6 times faster and covered an area 3 times larger when compared to the 5.0 mg/ml one (Fig. 3b). These findings suggest that physical interactions of the RGD4C.AAVP particles with the tumor microenvironment create significant obstacles for phage-guided gene transfer.

Next, we determined whether ECM in the microenvironment could be manipulated to increase cellular uptake of RGD4C.AAVP after treatment with the ECM-degrading enzymes. An RGD4C.AAVP internalization assay was performed by which the intracellular post-transduction RGD4C.AAVP was quantified by flow cytometry. Figure 3c depicts significant enhancement of RGD4C.AAVP endocytosis in 9L tumor cells following ECM removal compared to control treatment with RGD4C.AAVP alone (without enzymatic ECM depletion). Combination treatment with both collagenase and hyaluronidase allowed up to 37 % increase in RGD4C.AAVP internalization, reflected by higher intracellular RGD4C.AAVP signal as well as enhanced RGD4C.AAVP signal counts per 10,000 cells (Fig. 3d). Interestingly, ECM depletion did not affect the specificity of RGD4C.AAVP cancer cell internalization as no effect was observed on the control non-targeted NT.AAVP (Fig. 3c). These data demonstrate the role of the ECM as a physical barrier to successful RGD4C.AAVP entry into cancer cells.

**Fig. 4:**
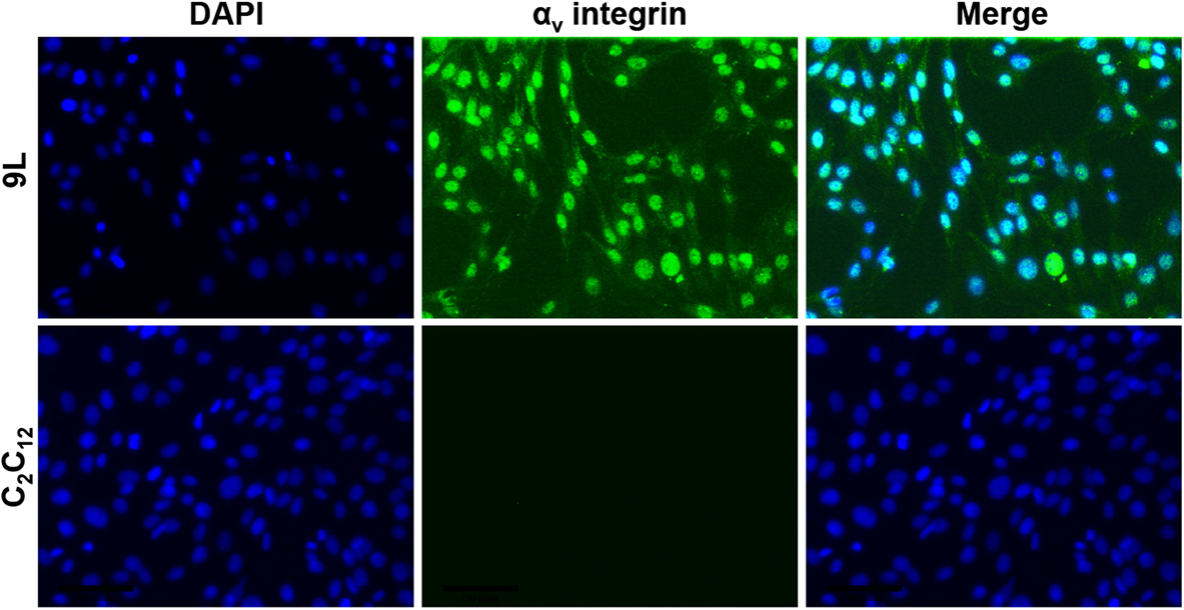
Immunostaining of α_v_ integrins in 9L cells. Cells seeded on coverslips were fixed and immunofluorescence stained using rabbit anti-α_v_-integrin primary and goat anti-rabbit AlexaFluor-488 secondary antibodies. The α_v_ integrin-associated fluorescence is shown in green and nuclei (DAPI) in blue. Experiments were repeated three times. The mouse C_2_C_12_ myoblasts were included in this experiment as negative control for α_v_ integrin expression

Because RGD4C.AAVP requires expression of α_v_ integrins on the target cell for successful binding and internalization, we performed immunostaining on 9L cells to confirm expression of this tumor specific integrin to allow endocytosis (Fig. 4). The mouse C_2_C_12_ myoblast cells were used as negative control, as they do not express α_v_ integrins [16]. The results show that 9L cells express α_v_ integrins, but not the C_2_C_12_ cells. Additionally, it has been previously demonstrated that all cell lines we used in this study express α_v_ integrins [17–22].

### ECM depletion significantly increases targeted gene transfer by RGD4C.AAVP

Having shown that ECM degradation increased the cell accessibility and entry of RGD4C.AAVP vectors in cancer cells, we sought to determine if enhanced endocytosis translates to increased gene expression. 9L cells were transduced with RGD4C.AAVP/*GFP* or RGD4C.AAVP/*Luc*, carrying the green fluorescent protein (*GFP*) or the firefly *Luc* reporter genes. Various ECM depleted conditions were tested including collagenase, hyaluronidase, or a combination of both enzymes.

**Fig. 5:**
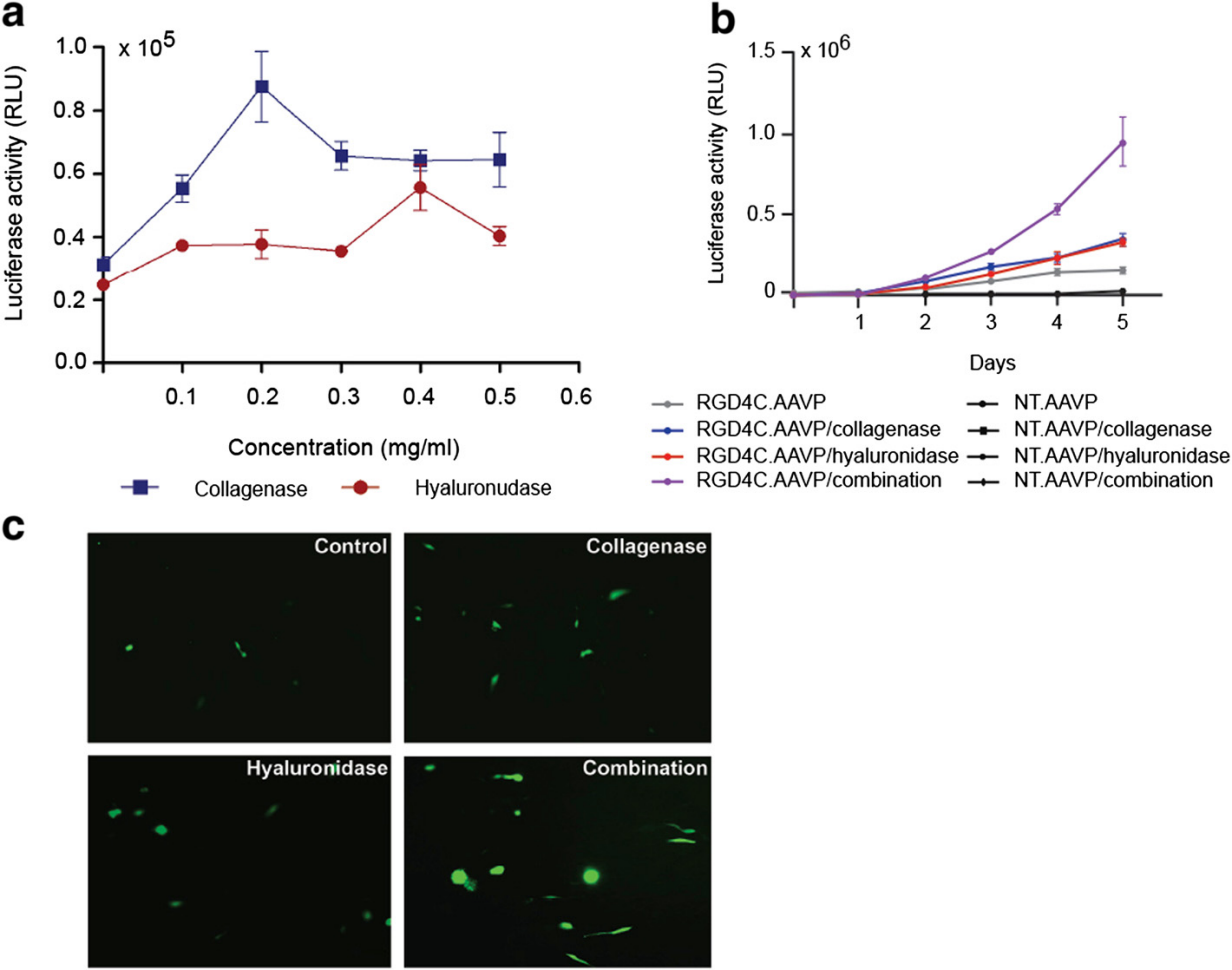
Characterization of the effect of ECM depletion on RGD4C.AAVP-guided gene transfer in 9L cells. **a** Luciferase expression in 9L cells by Steady-Glo® assay after treatment with increasing concentrations of collagenase or hyaluronidase, at day 3 post-transduction with RGD4C.AAVP/*Luc* vector carrying the *Luc* reporter gene. **b** Time course expression of luciferase over 5 days post transduction with RGD4C.AAVP vector alone, or RGD4C.AAVP in conjunction with collagenase (0.2 mg/ml) or hyaluronidase (0.4 mg/ml) or with combination of both enzymes. Similar enzymatic treatments were included with the control non-targeted NT.AAVP vector. **c** GFP expression in 9L cells transduced with RGD4C.AAVP-*GFP* alone (control) or following various ECM depletion strategies: collagenase, hyaluronidase or combination of both enzymes. Images were visualized by fluorescence confocal microscopy 3 days post vector transduction One way ANOVA was used, together with Tukey’s post-test to generate the data, *, p < 0.05, **, p < 0.01, ***, p < 0.001. Luciferase expression results are shown as relative luminescence units/1 μg protein. Samples were obtained through triplicate wells and each experiment was performed at least twice with consistent results. Data are presented as mean ± s.e.m. Experiments were repeated twice and individual conditions were repeated in triplicates

Firstly, quantification of gene expression was done using the RGD4C.AAVP/*Luc* vector 72 h post-transduction and a luciferase assay kit (Steady-Glo, Promega). To determine optimum concentrations of collagenase and hyaluronidase enzymes for use in future experiments, we carried out a titration experiment with increasing concentrations of both enzymes in 9L tumor cells (Fig. 5a). Levels of collagenase or hyaluronidase (0 mg/ml to 0.5 mg/ml) were tested for effects on RGD4C.AAVP-mediated *Luc* gene expression (Fig. 5a). In 9L cells, increasing collagenase levels resulted in enhanced gene expression by RGD4C.AAVP, peaking at 0.2 mg/ml and dropping at higher concentrations, whereas hyaluronidase application was most effective at 0.4 mg/ml (Fig. 5a).

It is also important to clarify whether enhanced gene expression is a transient or a long-term effect. We further determined in 9L cells, the changes in gene expression over time (5 days post vector transduction) through sequential luciferase assays (Fig. 5b). Differences in gene expression between the control RGD4C.AAVP alone and the combination enzyme treatment were significantly detectable from as early as day 2 after transduction, where a 1.9-fold higher *Luc* expression was detected in combination with ECM depletion by both collagenase and hyaluronidase enzymes (Fig. 5b). *Luc* expression in all groups continued to increase throughout the experimental timeframe and strong differences between treatment groups were clearly visible 5 days post transduction. Transgene expression levels in the combination treatment group reached a substantial 6.7-fold increase when compared to RGD4C.AAVP alone by day 5. Importantly, non-targeted NT.AAVP vector (lacking RGD4C ligand) was unable to achieve gene expression alone or in combination with ECM depletion (Fig. 5b) confirming that specificity is preserved when combining RGD4C.AAVP and ECM degradation.

Even though the biggest differences between treatment groups were clearly observed when transgene expression was allowed to run its course well past 72 h, further experiments were conducted with a 3-day limit to strike a balance between experiment turnover rate and any observable effects from ECM depletion. These experiments demonstrate that ECM degradation can result in long-lasting enhanced gene expression from the RGD4C.AAVP vector.

Finally, to qualitatively demonstrate the effect of ECM depletion on phage-guided gene transfer, we assessed GFP expression using RGD4C.AAVP/*GFP* vectors after enzymatic treatment (Fig. 5c). Based on the optimal enzyme concentrations found in Fig. 5a, confocal microscopic analysis of transgene expression 72 h post-transduction using RGD4C.AAVP/*GFP* vector in combination with ECM depletion supported the results obtained from the luciferase assay. As we expected, combination enzyme treatment at optimal concentrations resulted in an even higher GFP expression compared to that of each enzyme alone (Fig. 5c).

### Gene expression in 3D multicellular tumor spheroids (MCTS) of 9L cells

**Fig. 6:**
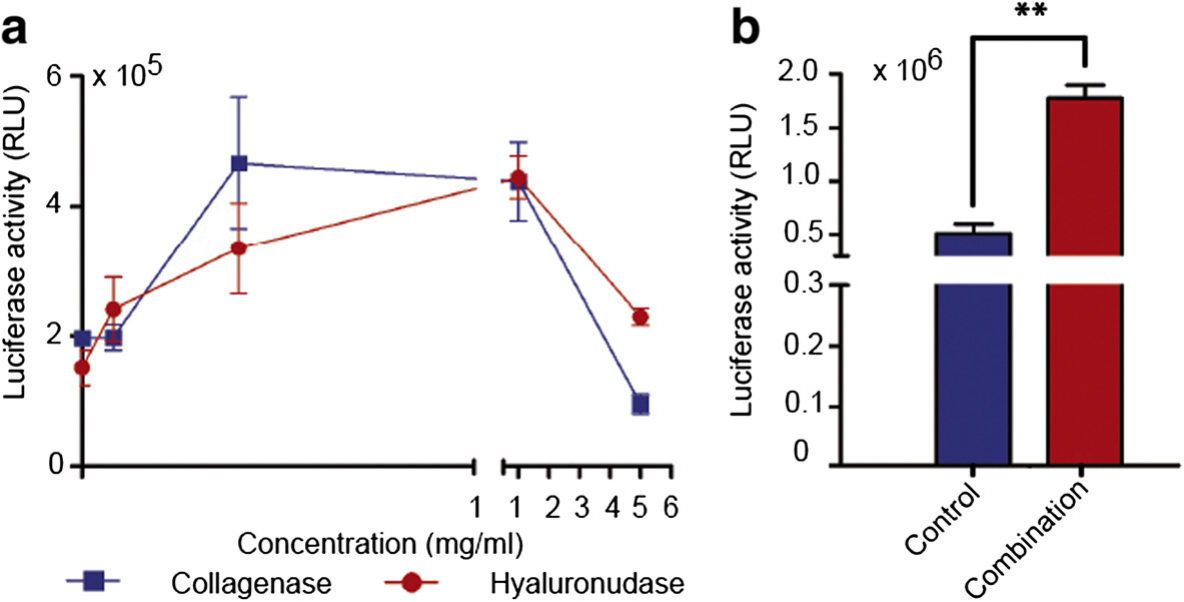
Multicellular tumor spheroid (MCTS) models showed increased targeted gene transfer by RGD4C.AAVP in combination with ECM depletion. **a** Concentration gradient curves of ECM degrading collagenase or hyaluronidase enzymes in 9L MCTS generated by quantification of post-transduction day 3 *Luc* gene expression through Steady-Glo® assay. RGD4C.AAVP/*Luc* was applied to cells that underwent collagenase or hyaluronidase treatments. **b** Quantification of RGD4C.AAVP-mediated luciferase expression in 9L MCTS following treatment with combination of optimal concentrations of collagenase (0.2 mg/ml) or hyaluronidase (1 mg/ml). RGD4C.AAVP/*Luc* alone, without enzymes, was included as control treatment. All experiments used triplicates for samples and were conducted at least twice. Luciferase expression results are shown as relative luminescence units/1 μg protein and presented as mean ± s.e.m.. One way ANOVA with Tukey’s post-test was used for the concentration curve whereas unpaired student’s *t*-test made comparisons between control and combination treatment, *, p < 0.05, **, p < 0.01, ***, p < 0.001. Experiments were repeated twice and individual conditions were repeated in triplicates

MCTS are increasingly recognized as superior tools over traditional 2D monolayer cell culture systems as they are able to mimic *in vivo* tumor microenvironements better [23]. Because tumor spheroids are much more complex than tumor cell monolayers, we postulated that the gene expression profile after treatment of ECM-depleting enzymes should be different. A range of collagenase or hyaluronidase concentrations (0 mg/ml, 0.04 mg/ml, 0.2 mg/ml, 1 mg/ml and 5 mg/ml) were tested on 9L MCTS followed by RGD4C.AAVP/*Luc* transduction and a luciferase assay (Fig. 6a).

Highest levels of luciferase expression were observed with 0.2 mg/ml of collagenase and 1 mg/ml of hyaluronidase with gene expression dropping in proportion to increase in enzyme concentration (Figs. 6a). These optimal concentrations were used in further tests combining both collagenase and hyaluronidase in treating 9L MCTS, showing a significant 2.6-fold increase of *Luc* transgene expression over control without ECM depletion (Fig. 6b).

### RGD4C.AAVP/*HSVtk* and GCV treatment in ECM depleted conditions results in increased tumor cell killing

To investigate whether increased transgene expression by RGD4C.AAVP in ECM depleted conditions could translate into enhanced cancer gene therapy, we incorporated in AAVP vectors the *HSVtk* cytotoxic gene, whose product activates the cytotoxic pro-drug ganciclovir (GCV). Addition of GCV to *HSVtk*-expressing cells induces cell death, as HSVtk expression results in formation of GCV-triphosphate, which is a chain terminating nucleoside analogue [24]. In these experiments, treatment with ECM-depleting enzymes was first carried out, then GCV treatment was initiated 72 h post RGD4C.AAVP transduction to allow sufficient *HSVtk* expression. Four cell lines were used to evaluate ECM depletion as a strategy to enhance therapeutic efficacy of RGD4C.AAVP, including 9L, M21 (human melanoma), U87 and SNB19 (human glioblastoma). Cell killing was assessed by quantification of dead cells using the trypan blue exclusion method.

**Fig. 7:**
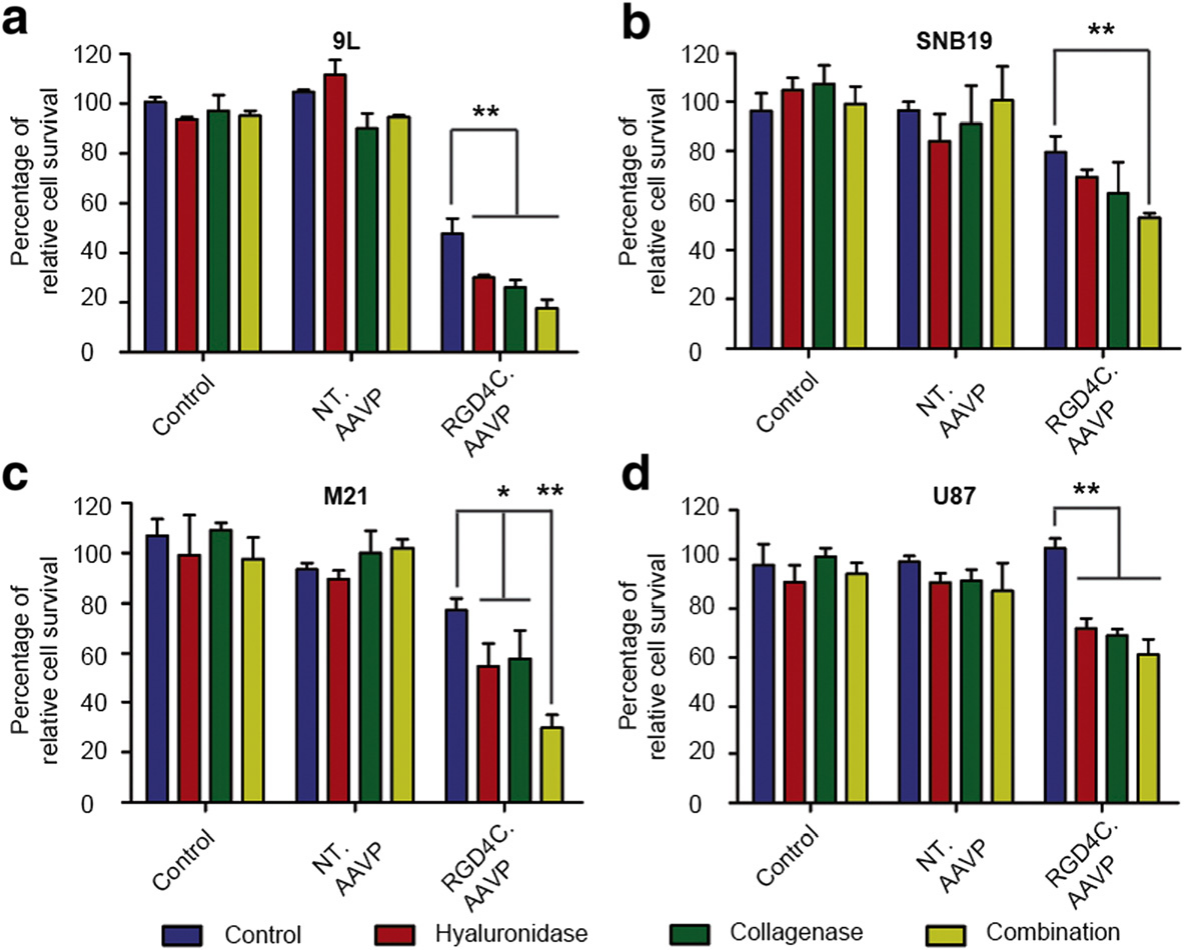
ECM depletion results in significant cell killing effects by RGD4C.AAVP-mediated *HSVtk*/GCV gene therapy. Cell viability of **a)** 9L rat gliosarcoma, **b)** SNB19 human glioblastoma, **c)** M21 human melanoma and **d)** U87 human glioblastoma measured after ECM treatment in conjunction with transduction by targeted RGD4C.AAVP or non-targeted NT.AAVP carrying the *HSVtk* transgene. The GCV (40 μM) was applied for 3 days and renewed daily starting 72 h after vector transduction. Cell killing was quantified by the trypan blue exclusion method and data expressed as percentage of control cells that were not treated with AAVP vectors. Data show the means of triplicate samples. A representative experiment is shown, and experiments were repeated three times with similar results. Statistics were performed by one way ANOVA with application of Tukey’s post-test, *, *p* < 0.05. Data are presented as mean ± s.e.m. Individual conditions were repeated in triplicates

We consistently observed a significant drop in cancer cell viability after combination enzyme treatment when compared to RGD4C.AAVP alone, across all four cell lines. 9L experienced the most significant drop by 63.0 % (Fig. 7a), followed by M21 (61.4 %, Fig. 7c), U87 (41.2 %, Fig. 7d) and SNB19 (33.2 %, Fig. 7b). In groups treated with either collagenase or hyaluronidase, a general drop in cell-viability was consistently observed when compared to RGD4C.AAVP alone, but was only significant for M21, 9L and U87 cell-lines. The decrease in cell-viability after treatment with either collagenase or hyaluronidase alone did not consistently produce significant drops compared to each other, but followed the general trend of decreased cell-viability compared to the group with no enzyme treatment.

### ECM depletion by enzymatic treatment does not affect cell viability

**Fig. 8:**
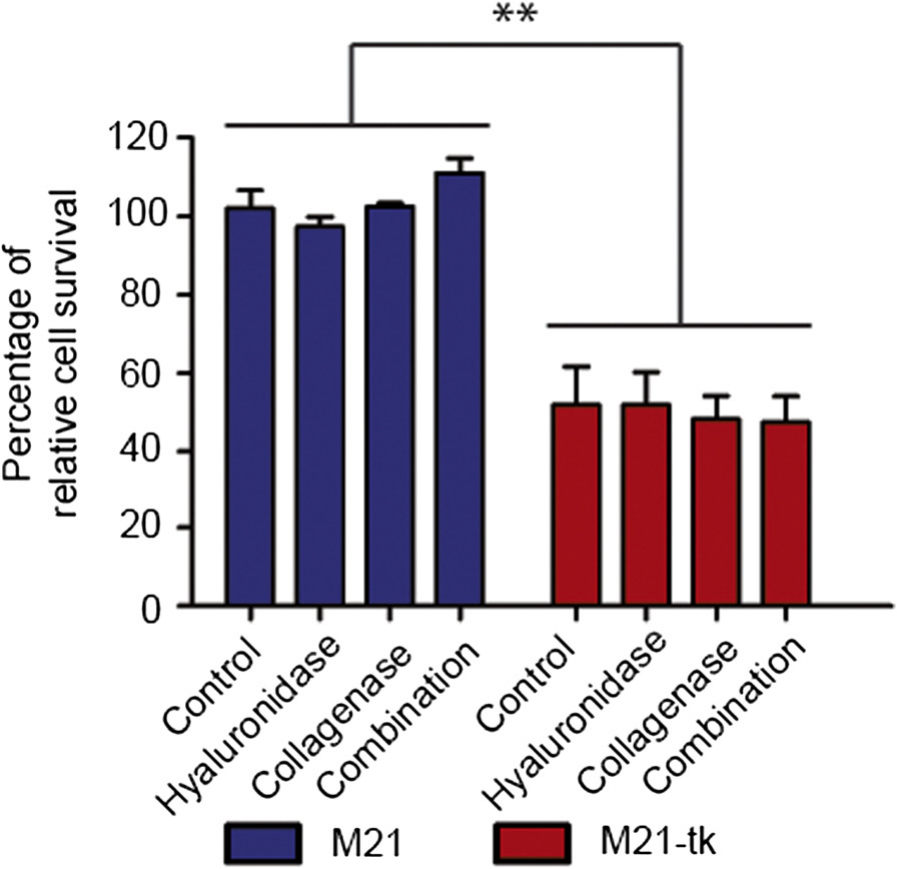
ECM depletion does not have an intrinsic effect on tumor cell viability. The M21 tumor cells were transduced with RGD4C.AAVP/*HSVtk-puro* ^*R*^ containing the *HSVtk* and a puromycin resistant gene***.*** At day 3 post vector transduction, cells were diluted and grown in puromycin- containing medium. At day 14 post vector transduction, M21 puromycin resistant cell clones were pooled as a population stably expressing HSVtk (M21-tk) and grown for a few weeks. Next, the M21-tk as well as parental M21 cells were subjected to enzymatic treatments with hyaluronidase or collagenase, or combination of both. GCV treatment started the next day and was carried out for 3 days. Evaluation of cell viability was performed by using the CellTiter Glo. Data show the mean of triplicate samples and are expressed as the percentage of parental M21 cells. A representative experiment is shown, and experiments were repeated twice with similar results. Statistics were performed by one way ANOVA with application of Tukey’s post-test, *, *p* < 0.05. Data are presented as mean ± s.e.m. Experiments were repeated twice and individual conditions were repeated in triplicates

We sought to verify that tumor cell viability is not affected by enzymatic treatment, in order to confirm that increased tumor cell killing by RGD4C.AAVP/*HSVtk* plus GCV is the result of increased *HSVtk* gene expression. Therefore, we established a stable cell population expressing HSVtk by transducing M21 cells with RGD4C.AAVP*/HSVtk* containing the puromycin selection gene (termed M21-tk). Next, the stable cell population was treated with collagenase, hyaluronidase or a combination of both enzymes at several weeks after the initial treatment with RGD4C.AAVP/*HSVtk*, to exclude the presence of RGD4C.AAVP particles. Treatment with GCV started 72 h after enzyme treatment. Using a cell viability assay, we observed a significant drop in cell viability in M21-tk by approximately 50 % compared to parental non-transduced M21 cells (Fig. 8). Importantly, there was no detectable effect of ECM depletion on the viability of parental M21 cells. There were no significant differences between cell viability across treatments groups of M21-tk cell population. Taken together, these data indicate that enzymatic treatments with collagenase or hyaluronidase or combination of both do not affect viability of cells and that differences in cell viability after RGD4C.AAVP therapy are due to improved vector diffusion through the ECM and subsequently enhanced tumor cell transduction by RGD4C.AAVP, but not enzymatic treatment.

### ECM depletion through losartan enhances transduction by RGD4C.AAVP

**Fig. 9:**
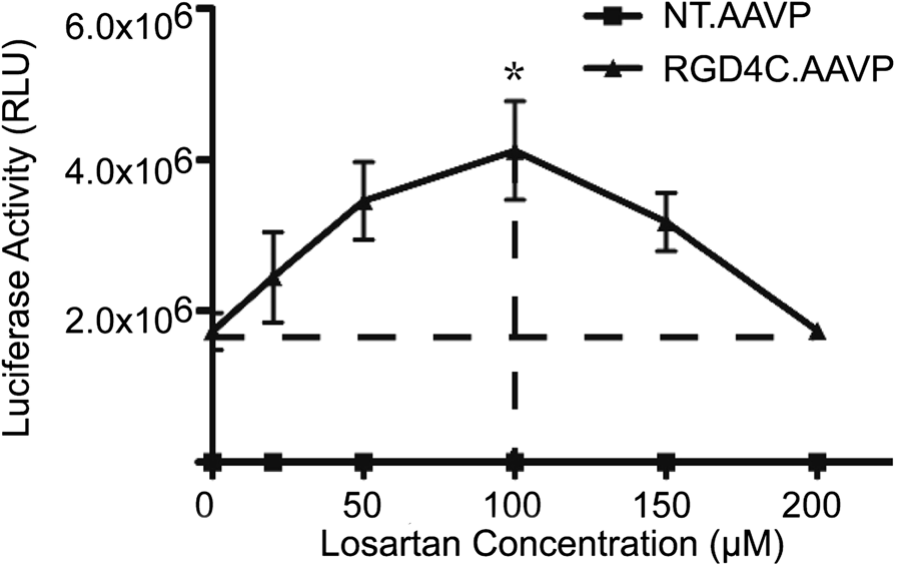
Losartan-mediated effects on RGD4C.AAVP transduction. 9L cells were incubated overnight with increasing concentrations 20 μM, 50 μM, 100 μM, 150 μM, 200 μM of losartan and transduced the following day with targeted RGD4C.AAVP or non-targeted NT.AAVP vectors expressing the *Luc* gene. *Luc* gene expression was quantified 3 days post-transduction by the luciferase Steady-Glo® assay kit. Means from triplicates are shown while the graph is representative of similar experiments (n = 2). One way ANOVA with application of Tukey’s post-test, *, *p* < 0.05, was performed to determine conditions for maximal transduction success rate. Data are presented as mean ± s.e.m. Experiments were repeated twice and individual conditions were repeated in triplicates

To determine whether ECM depletion through drug treatment will have similar effects on gene transduction by RGD4C.AAVP as enzyme treatment, we used losartan, a FDA-approved (Food and Drug Administration, US) antihypertensive that also inhibits collagen I synthesis, to deplete collagen in 9L cells [25]. Luciferase reporter assays were repeated to determine whether losartan could be a possible replacement for collagenase. When pre-treated with losartan at an optimal concentration (100 μM), the transduction efficiency of RGD4C.AAVP was significantly enhanced, peaking at 4.0 × 10^6^ relative luminescence units, which is approximately 2.4-fold higher than the control group with no losartan added (Fig. 9). Again losartan didn’t affect the tumor cell specificity of the RGD4C.AAVP since the control non-targeted NT.AAVP was unable to deliver gene expression in the presence of losartan.

## Discussion

In this study, we demonstrate the effectiveness of ECM depletion as a strategy to enhance bacteriophage-guided gene transfer to cancer cells. By removing various ECM constituents using collagenase, hyaluronidase or a combination of both, we were able to substantially increase RGD4C.AAVP internalization and RGD4C.AAVP-mediated gene expression, leading to enhanced therapeutic efficacy. The most dramatic effect was observed when various tumor cell lines were treated with a combination of both enzymes in both 2D and 3D MCTS settings.

The data provide evidence that a combination of spatial and biochemical changes are responsible for the increase in gene transfer efficacy following enzyme-induced ECM depletion. Because the tumor ECM is a primary obstacle that impedes therapy from reaching target cells, when ECM is depleted, the spatial distribution and adsorption of RGD4C.AAVP vectors on the tumor cell surface are improved. Furthermore, ECM constituents may also biochemically impede vector access to tumor cells. Two interesting ECM constituents that had the highest impact in terms of competitive inhibition are collagen and fibronectin; both of these constituents are ligands for α_v_ integrins which are also the receptor targets of the RGD4C ligand displayed on RGD4C.AAVP and substantially decreased following enzymatic treatment [26, 27]. Our data also show that hyaluronic acid was also released by enzyme treatment; however, it has not been shown that it may compete or interact with α_v_ integrin receptors. These data suggest that enhanced gene expression by RGD4C.AAVP vectors after enzyme-mediated ECM depletion is a function of both physical and biochemical changes in the ECM.

When judging the effect of ECM depletion on RGD4C.AAVP gene therapy, it is crucial to consider gene transduction at all stages. Unlike drug molecules, RGD4C.AAVP cannot easily diffuse through the ECM; it is a relatively narrow (6.5 nm width) elongated cylinder reaching up to 1400 nm length [28] making cellular access difficult in the presence of ECM. Additionally, the ECM is hydrophilic, subsequently increasing the relative volume of the microenvironment surrounding tumor cells.

Our experiments provide the first proof of concept evidence that ECM clearance can be used in phage-guided gene transfer to improve targeted cancer cell killing. As we have shown, collagenase and hyaluronidase are able to substantially increase RGD4C.AAVP-mediated gene transfer efficacy in cancer cells and exert additive effects when both are applied. These effects can be explained, as collagen and hyaluronic acid are separate ECM constituents that may inhibit vector transduction in various ways. However, high levels of enzymes, especially collagenase, seem to be counterproductive to transduction. We suspect this is likely due to physical loss of detached cells during washing, rather than a direct alteration of cellular function due to enzyme-treatment.

Administration of collagenase into tumor-bearing animal models has been proven to decrease the interstitial fluid pressure and improve gene therapy vector accessibility as well as transduction [29]. Despite FDA-approved use as a locally injected treatment in man [30], intravenous administration of collagenase remains a particular concern since they do not discriminate between healthy and diseased tissues. As a result, injections may cause tissue damage, as demonstrated by lung necrosis and hemorrhages in murine models [31]. Moreover, the ECM has been implicated in many cancer pathways including metastasis [32]. High levels of interstitial matrix metalloproteinases (MMPs), specially collagenase, have been found to correlate with poorer prognosis and increased metastatic potential for some cancers [33]. Therefore, the clinical translatability of ECM depletion as a strategy to enhance cancer therapy warrants further investigation.

One potential way of circumventing systemic ECM degradation is to use a pharmacological agent to inhibit its production. Losartan is a candidate angiotensin II type I receptor antagonist for hypertension, which has shown anti-fibrotic activity mediated by the Tumor Growth Factor Beta 1 pathway through thrombospondin-1 (TSP-1), leading to inhibition of collagen type I synthesis [34, 35]. Its combination with oncolytic HSV has been efficacious in murine xenograft models of human cancers, and has shown limited and manageable side effects in patients [14, 36], offering a better safety profile than intravenous collagenase. Losartan’s mechanism of action inhibits new synthesis of collagen rather than degrading collagen, meaning it may preferably affect more metabolically active cancer cells. Furthermore, losartan has potentially multiple anti-cancer properties such as metastatic suppression via TGF-β1 signaling [37] that make it especially attractive for use in this field. Thus, drug modulation of collagen may provide an alternative strategy for enhancing gene transfer in cancer. In our study we showed that losartan produced a significant gene expression increase by RGD4C.AAVP vector without affecting its tumor cell specificity.

While we observed changes in multiple ECM constituents, fibronectin is clearly affected after treatment with collagenase. Multiple studies have shown that fibronectin synthesis is positively related to progression and metastases of cancer [38]. Suppression of growth factor signaling pathways can down-regulate fibronectin synthesis, and is a potential strategy for enhancing penetration of vector/cell-based therapies to solid tumors [39–41]. Direct or indirect inhibition of fibronectin will significantly contribute to enhanced therapy, as it is a physical barrier to tumor cells, in addition to being competitive inhibitor of receptors that RGD4C.AAVP vectors use for tumor cell entry [26]. Our data suggest that down-regulation of fibronectin, combined with collagen, may significantly account for increased gene expression from the RGD4C.AAVP vector; indeed, this is supported by previously reported *in vivo* data for other gene therapy vectors [42]. Strategies to inhibit fibronectin synthesis or degrade existing tumor-associated fibronectin are crucial in the context of ECM depletion in gene therapy vectors, including RGD4C.AAVP.

Though we have focused on collagen and fibronectin as the main inhibitory ECM constituents, therapies involving hyaluronidase are also used in clinical settings. Intravenous hyaluronidase has already been used as a safe drug for myocardial infarction in man [43]. Different levels of hyaluronidase have been associated with both cancer growth and suppression with some studies showing tumor-associated production of the enzyme, whereas others have shown that higher concentrations inhibit tumorigenesis. This suggests the potential role of hyaluronidase as an adjuvant for breast cancer chemotherapy [44]. Though efficacious, it has not found widespread use due to its immunogenicity, as it is of bovine origin [45]. However, a recently FDA-approved recombinant human hyaluronidase (PEGPH20, Halozyme Therapeutics) is being evaluated in an ongoing trial for patients with advanced solid tumors [46, 47], suggesting that hyaluronidase can be a clinically feasible anti-cancer adjuvant.

Alternative strategies for decreasing hyaluronic acid production, such as enzyme inhibition have also been considered. Inhibition of hyaluronic acid synthase (*HAS*) using antisense oligonucleotides has been used in murine *in vivo* models to halt cancer progression [48]. Another small molecule HAS inhibitor, 4-methylumbelliferone has even shown anticancer effects independent of ECM depletion effects, consequently reducing proliferation and cellular metastatic potential [49].

We have also demonstrated the feasibility of ECM depletion in 3D MCTS, which are thought to be superior models for *in vivo* tumors [50]. The MCTS model reflects possible resistance to RGD4C.AAVP’s penetration to reach the target cells, due to their metabolic activities, physical characteristics (hypoxia and lack of vascularization) and differential regulation when compared to 2D models [23]. Because cell monolayers lack the complexities of 3D tumors, they are more susceptible to therapy in vitro. By demonstrating the effectiveness of ECM modulation in enhancing RGD4C.AAVP-meditaed gene transfer in an MCTS setting, we show that our strategy of ECM depletion is relevant for *in vivo* gene based therapies.

Our findings also establish the applicability of combining ECM depletion with *HSVtk*-GCV therapy. A prominent problem with cancer gene therapy is incomplete eradication of tumors, which frequently translates into cancer recurrence [51, 52]. ECM depletion allowed significant eradication of tumor cells by RGD4C.AAVP gene therapy, which demonstrates its potential as a therapeutic strategy for future studies.

## Conclusions

ECM depletion greatly enhances gene transfer of phage-derived vectors to deliver therapeutic genes specifically to cancer cells. We provide evidence for how the ECM plays physical and kinetic roles in impeding successful cancer cell binding and entry of RGD4C.AAVP and subsequent gene transfer by this phage vector, and potentially other virus-based delivery strategies. By eliminating the external barrier of tumors and increasing vector transduction at multiple stages, this strategy may improve the penetration of gene therapy vectors into solid tumors. With many biologically safe inhibitors and ECM-depleting enzymes becoming available, this strategy warrants further clinical investigation. Here, we provide evidence that ECM depletion is a clinically translatable strategy with great potential to enhance RGD4C.AAVP-guided cancer therapy.

## Material and methods

### Cell culture

Rat 9L gliosarcoma cells were a gift from Dr Hrvoje Miletic (University of Bergen, Norway) and human M21 Melanoma cells were provided by Dr David Cheresh (University of California, La Jolla). Human LN229 and SNB19 glioblastoma cells were provided by Dr Nelofer Syed (Imperial College London, UK), LNCaP prostate cancer cell line was a gift from Dr Paul Mintz (Imperial College London) and the mouse C_2_C_12_ myoblast cell line was provided by Dr Francesco Muntoni (University College London, UK). The human breast cancer MCF-7 and glioblastoma U87 cell lines were from Cancer Research UK. Cells were sustained in Dulbecco’s Modified Eagle’s Medium (DMEM, Sigma) supplemented with 10 % Fetal Bovine Serum (FBS, Sigma), L-Glutamine (2 mM, Sigma), Penicillin (100 units/ml, Sigma) and Streptomycin (100 μg/ml, Sigma). The C_2_C_12_ cells were grown in 20 % FBS. Cells were maintained at 37 °C in a humidified atmosphere supplemented with 5 % CO_2_. Collagenase from *Clostridium histolyticum* (Type I, ≥125 CDU/mg, Sigma) and hyaluronidase from bovine testes (Type I-S, 400-1000 units/mg, Sigma) were solubilized in phosphate buffered saline (1x PBS, Sigma).

### Immunostaining

Immunostaining experiments were performed as previously described [28]. Brief, cells were seeded on 18 mm^2^ coverslips in 12-well plates and allowed to proliferate until 70-80 % confluence. Cultures were then treated with enzymes as indicated, washed with PBS, and fixed in 4 % PFA in PBS for 10 min at room temperature. Next, cells were quenched with 50 mM Ammonium Chloride (NH_4_Cl), washed with PBS and blocked with PBS containing 2 % bovine serum albumin (BSA) for 30 min. Cells were incubated with mouse anti-fibronectin or anti-laminin antibodies (BD Biosciences) at 1:200 dilution overnight at 4 °C, or 1 h at room temperature with a rabbit anti-α_v_ integrin (diluted 1:50). Then, cells were incubated with secondary AlexaFluor-conjugated antibodies (diluted 1:750) and with 4',6-diamidino-2-phenylindole (DAPI) (diluted 1:2000 in 1 % BSA-PBS) for 1 h at room temperature. Finally, cells were mounted in Prolong Gold antifade mounting medium (Invitrogen) and images were obtained using a fluorescent microscope (Nikon Eclipse TE2000U) and analysed by Openlab imaging software.

### Hyaluronic acid (HA) ELISA

HA ELISA was performed as previously described [53]. Briefly, samples or standard umbilical cord HA (Sigma) at various concentrations (19–10,000 ng/ml) in PBS pH 7.4, were added to 1.5 ml plastic tubes containing biotinylated hyaluronic acid binding protein (HABP, 1:200 in 0.05 M Tris–HCl buffer, pH 8.6). The tubes were incubated at room temperature for 1 h, then samples were added to the microplate, which was pre-coated with umbilical cord HA (100 μl/well of 10 mg/ml), and blocked with 1 % BSA (150 μl/well). The plate was then incubated at room temperature for 1 h. The wells were subsequently washed and 100 μl of peroxidase-conjugated anti-biotin antibody (Sigma) were added. The plate was incubated at room temperature for another hr. The reaction was stopped with 50 μl/well of 4 M sulfuric acid and the absorbance was determined using a microplate reader (Molecular Devices) at 492/690 nm. The concentration of HA in samples was calculated by reference to a standard curve.

### Collagen depletion assay (Sirius Red)

Collagenase from *Clostridium histolyticum* (Type I, ≥125 CDU/mg, Sigma) was solubilized in 1x PBS. Tumor cells were seeded in 12-well plates at a density of 120,000 cells/well and cultured for 72 h until confluence. Cells were then washed with PBS and 500 μl of 0.2 mg/ml collagenase in serum free media was added to the cells and left to incubate at 37 °C for 1 h. Next, both cells and supernatant were processed as different samples in a collagen I detection assay. The assay was conducted based on the use of the Sirius Red dye and by following the manufacturer’s instructions of a Sirius Red Total Collagen Detection Kit (Chondrex, Inc.), with final measurements obtained through a Promega plate reader.

### AAVP diffusion assay

200 μl of ECM Gel from Engelbreth-Holm-Swarm murine sarcoma (Sigma) at 2.5 mg/ml or 5.0 mg/ml were added to a 48-well plate and allowed to set at 37 °C. In the meantime fluorescently-tagged RGD4C.AAVP was prepared at a concentration of 5 μg/ml. 50 μl of the RGD4C.AAVP solution were taken up in a pipette tip, which was inserted at a fixed position into the ECM-gel matrix and left to diffuse through the material for 1 h. Measurements were obtained at 1 h post diffusion and at 30 min intervals thereafter.

### AAVP production and purification

AAVP bacteriophages were generated as previously reported [54], by inserting an AAV mammalian viral gene transfer cassette into the fUSE5 plasmid derived from the fd-tet bacteriophage. Reporter or therapeutic genes were carried by the cassette, driven by a Cytomegalovirus (CMV) promoter. Targeting and specificity were achieved through genetic manipulation and display of the RGD4C peptide on the pIII minor coat protein conferring tumor-targeting properties [54]. AAVPs were produced and purified from the culture supernatant of *Escherichia Coli* K91 host bacteria as previously reported [54]. Isolated AAVPs were sterile-filtered through 0.45 μm filters and titration levels were quantified using a photospectrophotometer (NanoDrop, Thermo Scientific).

### Generation of fluorescently-labelled RGD4C.AAVP

RGD4C.AAVP phage particles (1x10^13^) were conjugated to fluorescein isothiocyanate (FITC) for 1 h at room temperature in the dark [55]. Then the phage was purified by three consecutive polyethyleneglycol precipitations, titrated as described above. Phage labelling was confirmed by double-staining using a rabbit anti-phage antibody (Sigma). We have previously confirmed that the fluorescently-labelled phage retains target specificity and cancer cell transduction (unpublished data).

### AAVP internalization

Internalization assay was carried out as previously described [28]. After cell treatment with optimized concentrations of the enzymes, cells were treated with vectors for 4 h at 37 °C, then placed on ice to stop endocytosis and washed 3 times with PBS to remove unbound vectors. Surface bound vectors were removed by trypsinization after which cells were pelleted by centrifugation at 2000 rpm for 5 min and fixed in 4 % paraformaldehyde (PFA) for 10 min at room temperature. Untreated cells were used as negative controls. To detect internalised phage-derived vectors, cells were blocked with 0.1 % saponin in 2 % bovine serum albumin in PBS (BSA-PBS) for 30 min followed by staining with rabbit anti-phage antibody (diluted 1:1000) in 0.1 % saponin in 1 % BSA-PBS for 1 h at room temperature. Cells were pelleted and re-suspended three times in 0.1 % saponin in 1 % BSA-PBS, then incubated with goat anti-rabbit AlexaFluor-647 (diluted 1:500) for 1 h at room temperature. Finally, cells were washed twice with 0.1 % saponin-PBS and re-suspended in PBS before analysis.

Fluorescence-activated cell sorting (FACS) analysis was carried out using a FACscalibur Flow cytometer (BD Biosciences) equipped with an argon-ion laser (488 nm) and red-diode laser (635 nm). The mean fluorescence intensity was measured for at least 10,000 gated cells per triplicate well. Results were analysed using Flojo (TreeStar) software.

### AAVP transduction

Transduction of cells with vectors were carried out as we previously described [54]. Brief, 10,000–20,000 cells/well were seeded in 48-well plates and cultured for 48-72 h to achieve 70–80 % confluence. The cells were washed, then collagenase or hyaluronidase or combination of both enzymes in serum free media were added (as indicated) to the cells for 1 h at 37 °C before the enzyme-containing medium was removed. 110 μl of serum-free media with AAVP (as indicated) was then applied followed by incubation at 37 °C for 4 h, 400 μl of complete medium were subsequently added to the cells and left for 72 h at 37 °C. For transduction using RGD4C.AAVP/*HSVtk*, DEAE.DEXTRAN (60 ng/ 1 μg AAVP) was used to enhance gene transduction in line with our previously established protocol and findings [16]. The cationic polymer does not affect cell viability or specificity of the RGD4C.AAVP vector [16].

### Determination of tumor cell killing *in vitro*

Cells were seeded in 48-well plates and incubated for 48 h to reach 80 % confluence. Then, cultures were treated with collagenase or hyaluronidase enzymes or combination of both, as indicated. Enzyme solutions were removed from cultures and washed with PBS before treatment with AAVP vectors carrying the Herpes Simplex Virus-thymidine kinase (*HSVtk*) gene. Ganciclovir (GCV, Sigma) was added to the cells (40 μM) at day 3 post vector transduction and renewed daily. Viable cells were monitored under microscope and cell viability was measured at day three post GCV treatment by using the trypan blue exclusion method (Sigma). When treating the M21-tk cell population stably expressing the HSVtk gene, GCV was added to cells the next day following enzymatic treatments and renewed daily for 3 days.

### Reporter gene assays

Quantification of luciferase expression from cells transduced with AAVP-*Luc*, was performed as described [16]. Medium was aspirated before the addition of 110 μl of Glo Lysis buffer, 1x (Promega). 50 μl of the cell lysate was then transferred to a 96-well white opaque microplate (BD Falcon) and mixed with an equal volume of Steady-Glo® luciferase substrate (Promega) for luciferase and read with a Promega Glomax plate reader. Luciferase expression was normalized to 1 μg protein levels determined by the Bradford assay (Sigma) and data presented as relative luminescence units (RLU). GFP expression was visualized using a Nikon Eclipse TE2000-U fluorescence microscope, as were morphological observations of cell monolayers.

### Cell viability assays (CellTiter Glo)

Cells were seeded, treated and transduced in line with the cell viability assay for trypan blue exclusion method. However, a bioluminescence assay for cell viability (CellTiter Glo, Promega) was used according to the manufacturer’s instruction. The CellTiter Glo assay is a luciferase-based assay to measure cell viability based on available adenosine triphosphate in the cell lysate.

### Multicellular tumor spheroids (MCTS) culture and treatment

MCTS were seeded in 96-well ultra-low attachment surface plates (Corning) from trypsinized monolayer cultures at an optimized cell suspension density [56] of 0.5 x 10^4^ cells/ml in 200 μl. They were then incubated for 48 h at 37 °C prior to any treatment. Media was completely removed before the application of 110 μl of enzyme/serum-free medium mixture and left to incubate at 37 °C for 1 h. The enzyme-supplemented medium was then removed and the spheroids were washed with PBS before transduction with AAVP/*Luc* vectors (in 110 μl of serum-free medium) for 4 h at 37 °C. Following transduction, 100 μl of complete media were added to make up a total volume of 200 μl and the cells were left to develop gene expression over a 72 h incubation period at 37 °C.

For MCTS luciferase assays, media was removed and 15 μl of Glo Lysis buffer was added to one spheroid per well. The MCTS were then incubated for 30 min. 10 μl of cell lysate/well from each spheroid per experimental condition were taken from 4 wells and combined to make up 40 μl of total volume. This was mixed in a 1:1 ratio together with the Steady-Glo® luciferase substrate and left 10 min before measurement in the Promega plate reader. The luminescence signal was then normalized to 1 μg protein levels determined by the Bradford assay.

### Losartan treatment

Various losartan concentrations of 0 μM, 20 μM, 50 μM, 100 μM, 150 μM and 200 μM were applied in serum-free media to cells and left overnight in an incubator at 37 °C. The following day, losartan-containing media was removed and cells were transduced with vectors as described above. Luciferase assays were carried out at 72 h post-transduction.

### Statistical analyses

Data are expressed as mean ± standard error of the mean (s.e.m.) and analysed using student’s *t* test when comparing two groups. To compare more than two groups, we used one-way ANOVA and *post hoc* Turkey tests. *P* values were considered significant when <0.05 and denoted as follows: *p < 0.05, **p < 0.01, ***p < 0.001. Statistical analyses were performed using the GraphPad Prism software (version 5.0).

## Abbreviations

AAV: Adeno-associated virus
AAVP: Adeno-associated virus/phage
CMV: Cytomegalovirus
ECM: Extracellular matrix
FBS: Fetal Bovine Serum
GCV: Ganciclovir
GFP: Green fluorescent protein
Luc: Luciferase
MCTS: Multicellular tumor spheroid
PBS: Phosphate buffered saline
RGD: Arginine-glycine-aspartic acid
